# Addicted to cellphones: exploring the psychometric properties between the nomophobia questionnaire and obsessiveness in college students

**DOI:** 10.1016/j.heliyon.2018.e00895

**Published:** 2018-11-01

**Authors:** Seungyeon Lee, Minsung Kim, Jessica S. Mendoza, Ian M. McDonough

**Affiliations:** a The University of Arkansas at Monticello, School of Social and Behavioral Sciences, 562 University Drive, Monticello, Arkansas 71656, USA; b Buros Center for Testing, University of Nebraska at Lincoln, 21 Teachers College Hall, Lincoln, Nebraska 68588-0348, USA; c The University of Alabama, Department of Psychology, 505 Hackberry Lane, Box 870348, Tuscaloosa, Alabama 35487, USA

**Keywords:** Psychology

## Abstract

A potential new clinical disorder is arising due to the addiction to cellphones called nomophobia—or feelings of discomfort or anxiety experienced by individuals when they are unable to use their mobile phones or utilize the conveniences these devices provide. However, before being able to officially classify this disorder as clinically relevant, more research needs to be conducted to determine how nomophobia relates to existing disorders. In a sample of 397 undergraduate students, the present study examined the relationship between the Nomophobia Questionnaire (NMP-Q) and the Obsessiveness Content Scale (OBS) of the Minnesota Multiphasic Personality Inventory-2 (the MMPI-2). Confirmatory factor analysis (CFA) was used to test whether the OBS Content Scale would be related to a one-factor NMP-Q solution (Fig. 1) or a four-factor NMP-Q solution (Fig. 2). Convergent and divergent validity were also investigated. The four-factor model was a better fit than the one-factor model as indicated by most fit indices. The findings showed that the OBS latent variable was correlated with all of the four NMP-Q latent variables. Mixed support was found for convergent validity, but high support was found for the divergent validity of the NMP-Q factors. This study contributes to a growing body of literature seeking to better understand the addictive nature of cellphones and takes a new perspective on addiction research and obsessiveness. These findings provide a better understanding between pre-existing assessments of personality disorders (e.g., obsessiveness) that are emerging from the overuse of mobile phones or the excessive fear of losing one's cell phone.

## 1 Introduction

Advances in technology exert a constantly changing effect on people's lifestyle. For example, mobile technology has enhanced people's access to information globally ever since wireless service was made available to the general public (King et al., 2013; Lee et al., 2014). Frequent technology usage also reinforces habitual checking of texts and social media outlets. This pattern of excessive smartphone use has potential short-term negative consequences such as distraction in the classroom and increased levels of anxiety (Cheever et al., 2014; Dietz and Henrich, 2014; Lee et al., 2017; Mendoza et al., 2018; Ravizza et al., 2014; Ruston et al., 2017; Thornton et al., 2014). More importantly, these technological developments also have the potential for long-term consequences by influencing personality disorders and may even exacerbate existing personality disorders (e.g., obsessive-compulsive personality disorder, social interaction anxiety, internet or smartphone addiction, etc.) (Bragazzi and Del Puente, 2014). These issues have led to the creation of several new measures assessing the influence of technology on behaviors (Bragazzi and Del Puente, 2014; Lin et al., 2014; Yildirim and Correia, 2015; Yildirim et al., 2016). Given the recency of these new measures, independent tests of questionnaire validity still remain open to be explored. Furthermore, little research has investigated the degree to which these new measures relate to well-validated and pre-existing measures that identify psychological disorders (i.e., convergent validity).

One common technology that has pervasively entered people's lives is the mobile phone. Recent data from the Pew Internet and American Life Project found that approximately 91% of people in the United States own and frequently use a mobile phone (Smith and Page, 2015). Frequent (and perhaps compulsive) usage of a mobile phone is positively correlated with mobile phone addiction, anxiety, depression, and stress (Bianchi and Phillips, 2005; Billieux et al., 2015; Lee et al., 2014; Lin et al., 2014; Nikhita et al., 2015; Roberts and Pirog, 2013; Takao et al., 2009; Thomée et al., 2011). Excessive mobile phone use has emerged as a recognizable social problem and will likely become more serious with its growing popularity.

In fact, being without one's mobile phone can have such dramatic effects on one's psychological well-being that a new term was recently coined to describe the anxieties of losing or being temporarily without one's mobile phone—*nomophobia* (Bragazzi and Del Puente, 2014; Dixit et al., 2010; King et al., 2010; King et al., 2013; King et al., 2014; Lee et al., 2014; Yildirim and Correia, 2015; Yildirim et al., 2016). Nomophobia is an abbreviated form of “no mobile phone phobia” and is thought to stem from the excessive use of a mobile phone. Individuals who are highly nomophobic tend to excessively check text messages or social media and have difficulty paying attention to daily tasks because they are afraid of losing connectedness and/or the ability to access information (Yildirim and Correia, 2015; Yildirim et al., 2016).

## 2 Background

Research conducted by King and collaborators (2010, 2013, 2014) conceptualize nomophobia as a modern “phobia.” However, the terms phobia and addiction have been interchangeably recognized throughout many studies when introducing the common symptoms of nomophobia (Alosaimi et al., 2016; King et al., 2013, 2014; Lee et al., 2014; Nikhita et al., 2015; Yildirim et al., 2016). These studies have suggested that in addition to fear-like symptoms, the excessive mobile phone has been closely associated with the notions of addiction, compulsiveness, and anxiety. Notably, some theorists argue that nomophobia is a misused term and can be better interpreted as a type of anxiety, addiction, or behavioral disorder rather than a fear, per se (Bianchi and Phillips, 2005; Loren, 2012). Despite the many interpretations, nomophobia is usually considered a “situational phobia” (King et al., 2010, 2013, 2014; Lin et al., 2014; Yildirim and Correia, 2015; Yildirim et al., 2016). Still, several studies have shown that phobias, addictions, and anxieties are not isomorphic with each other (Binanchi and Phillips, 2005; Lee et al., 2014; Loren, 2012). It also might be the case that nomophobia resembles the behavior of previously mentioned personality disorders, particularly obsessive compulsiveness. However, because the term nomophobia is relatively new to the field of clinical psychology (Arpaci, 2017; Bragazzi and Del Puente, 2014; King et al., 2010; King et al., 2013; King et al., 2014; Yildirim et al., 2016), few studies have examined the relationship between nomophobia and other types of mental disorders such as obsessive-compulsiveness. This study seeks to examine the shared traits in obsessiveness and nomophobia as obsessiveness is a trait that has been linked to other disorders such as eating disorder, depression and substance addiction (Bragazzi and Del Puente, 2014). Understanding what factors of nomophobia, if any, could be useful in the identification to disorders that are comorbid.

## 3 Theory

The nomophobia questionnaire (NMP-Q) was recently developed to assess the dimensions of nomophobia (Yildirim and Correia, 2015). In the questionnaire's development, Yildirim and Correia (2015) described four factors of the NMP-Q using Explanatory Factor Analysis (EFA). A principal component analysis (PCA) approach with varimax rotation was performed to examine the correlations among the items. The authors concluded that the use of PCA was appropriate since correlations among the items exceeded 30 (Tabachnick and Fidell, 2013; Yildirim and Correia, 2015). From this PCA, the four factors were “not being able to communicate,” “losing connectedness,” “not being able to access information,” and “giving up convenience.” Cronbach's alpha coefficient was 0.95 overall, and for the four different factors, the alphas were 0.94, 0.87, 0.83, and 0.81 respectively. Yildirim and Correia (2015) concluded that the NMP-Q had good internal consistency and was fairly reliable.

As Yildirim and Correia (2015), EFA was conducted. Their analysis showed that a Cronbach's alpha coefficient was 0.95 overall, but three rather than four factors best described the structure of the questionnaire with a Cronbach's alpha for the three factors being 0.94, 0.89, and 0.88. To test for content validity, researchers referred to two experts who reviewed the survey for the clarity, importance, and relevance of the items in the questionnaire. Next, researchers tested for construct validity by correlating the NMP-Q with a relevant measure, the Mobile Phone Involvement Questionnaire (MPIQ) (Walsh et al., 2010). A product-moment correlation revealed that there was a strong correlation between the scores from the MPIQ and the NMP-Q, *r* (299) = .710, *p* < .01. This correlation demonstrated a strong similarity between the two scales, and thus the authors concluded that the NMP-Q is fairly reliable.

Similarly, Bragazzi and his collaborators (2016) psychometrically validated an Italian version of the NMP-Q on a sample of 403 participants. Researchers recruited data by convenience sampling and casted the questionnaire online among young college students. The questionnaire was translated to Italian using a “backwards and forwards” approach meaning that the questionnaire was translated from English to Italian while focusing on the conceptual meaning of a set of words or phrases rather than the literal translation. Researchers gathered data from 403 participants who completed the Italian version of the NMP-Q along with a general questionnaire that included demographic information and average smartphone use. Findings demonstrated a Cronbach coefficient of .95 which was comparable to that of the original instrument, .945. Researchers also observed an association between the NMP-Q scores and the number of hours spent using a mobile phone which strengthened the validation to the Italian version of the NMP-Q. Furthermore, the large sample size provided more precision to the results that were found. Other than the validation of the development of the NMP-Q (Yildirim and Correia, 2015) and the validation of the translated version of the questionnaire (Bragazzi et al., 2016), some authors conducted Confirmatory Factor Analysis (CFA) to examine the relationship between the NMP-Q and other psychometric measures (Arpaci, 2017; Arpaci et al., 2017; Lin et al., 2018). Lin and Pakpour, 2017 found the concurrently validity of their study revealed that the NMP-Q is more likely to be correlated with the scales assessing similar psychological symptoms (i.e., anxiety) than symptoms that are different (i.e., hyperactivity). Still, not many studies have used a CFA approach to verify the NMP-Q as a means of understanding the addictive nature of cellphones. One of the few studies that examined addictive behavior and cellphones argued that addictive behaviors were attributed to coping mechanisms that are a part of common symptomology related to mental health adversities such as post-traumatic stress disorder (PTSD) (Contractor et al., 2017). Contractor et al. (2017) used a CFA approach to draw latent-level associations between PTSD symptom clusters and problematic smartphone use. The findings were aligned with other research that has supported excessive cellphone use with worsened feelings of anxiety (Lepp et al., 2013). The findings in the study conducted by Contractor and colleagues (2017) also are consistent with other research that suggests excessive cellphone usage to be a distraction-based coping mechanism to alleviate unpleasant feelings for individuals experiencing trauma and physiological arousal (Baker et al., 2004). Furthermore, researchers speculate that the problematics smartphone impulse may be more likely to occur in individuals who experience negative altercations in cognitions and mood (Contractor et al., 2017). Here, we propose a reinforcement of the perspective of excessive use of smartphones how this usage might be related to other research on addiction and obsessiveness. We specifically look at obsessiveness as a psychopathological interest since younger generations appear to be the population most affected by mobile devices to the point that it interferes in aspects of their daily life (Bianchi and Phillips, 2005; Lee et al., 2014, 2017; Yildirim and Correia, 2015).

The present study also used a CFA as an innovative psychometric method for investigating the psychometric properties of the NMP-Q. Our study provides additional evidence using both convergent and divergent validity analyses. To assess convergent validity, two common indices of all the measured constructs were used: Average Variance Extracted (AVE) and Composite Reliability (CR). These assessments test whether the constructs share sufficient variance to be considered a factor. In addition, the divergent validity of four NMP-Q factors (Yildirim and Correia, 2015) was examined to provide evidence that four factors of NMP-Q were discrete.

Given this context, nomophobia appears to be related to obsessiveness, but the relationship between the two have not been empirically examined. Recognizing fundamental symptomology may lead to a better understanding of people with nomophobia. To the extent that a large amount of shared variance is found between the two constructs, nomophobia might be best conceptualized as a specific manifestation of general obsessive tendencies. Conversely, a low amount of shared variance would suggest that nomophobia consists of discrete features. Second, we compared model fits of the two-factor model and a five-factor model that included four previously described dimensions of the NMP-Q. If the five-factor model fit better than the two-factor model, it would suggest that obsessive tendencies with the psychometrically validated measure are strongly correlated with some, but not all aspects of nomophobia.

## 4 Methods

### 4.1 Participants

Participants (*N* = 400) were recruited from undergraduate psychology courses using the psychology subject pool of two universities in Southeastern Arkansas and Alabama. The participants from southeastern Arkansas (n = 160) were recruited as a part of a different study investigating cell phone policies in the classroom (Lee et al., 2017). That study did not report associations between any of the measures used here. Participants included 130 men and 267 women, and one did not respond. Three participants (0.75%) had missing information and were excluded from the dataset. Participants received partial course credit for their completion of the study. The age (*M* = 20.69 and *SD* = 4.33) of the final 397 students ranged from 18 to 55.

## 5 Instrumentation

### 5.1 OBS content scale

The Obsessiveness (OBS) Content Scale of the Minnesota Multiphasic Personality Inventory-2 (MMPI-2) was designed to identify individuals with obsessive thoughts using 16 dichotomous items by Butcher and his collaborators (1989). Butcher et al. (1989) measured an internal consistency of the scale and found that Cronbach's alpha was .82, which is considered as fairly reliable. Two OBS Content Scale items having item-total correlation below .30 were excluded from the analysis of this study due to those poorly discriminating function. OBS Content Scale latent variable in the result section is from the MMPI-2. Cronbach's alpha of the present study was .72.

### 5.2 NMP-Q

The Nomophobia Questionnaire was used to measure nomophobia (Yildirim and Correia, 2015). The NMP-Q consists of 20 items that cover four main dimensions of nomophobia: not being able to communicate, losing connectedness, not being able to access information, and giving up convenience. Each item is measured by a 7-point Likert scale, with 1 being “strongly disagree” and with 7 being “strongly agree.” The NMP-Q has a Cronbach's reliability of .945 (Yildirim and Correia, 2015). Cronbach's alpha of the present study was .94 and for the four subscales was .92, .87, .85, and .83 respectively. No problematic univariate outlier was observed while observing interquartile ranges of six factors (NMP-Q, NMP-Q_F1-4, and OBS).

### 5.3 Procedure

All sessions were conducted in a classroom setting; the participants had 40 minutes to complete the measurements.

This study was conducted in accordance with the Declaration of Helsinki and the APA ethical standards, all participants were consented before participation, and was approved by the IRB boards of University of Arkansas at Monticello and the University of Alabama, Tuscaloosa.

## 6 Analysis

We used confirmatory factor analysis (CFA) to test whether a two-factor model that separated NMP-Q and OBS Content Scale was an adequate fit. CFA using Mplus 7.0 (Muthén and Muthén, 1998) was conducted to provide support for the construct validation of the two constructs and the relationships between them. CFA was chosen instead of EFA because both a theoretical framework and an existing model proposed through empirical research were given (Yildirim and Correia, 2015). Conventional measures of goodness-of-fit were used including the χ^2^ statistic, the root mean squared error of approximation (RMSEA), the standardized root mean square residual (SRMR), and the comparative fit index (CFI). Brown (2006) proposed three good model fit criteria: (1) below 0.08 for SRMR, (2) below 0.06 for RMSEA, (3) above 0.90 for CFI and TLI.

**Fig. 1:**
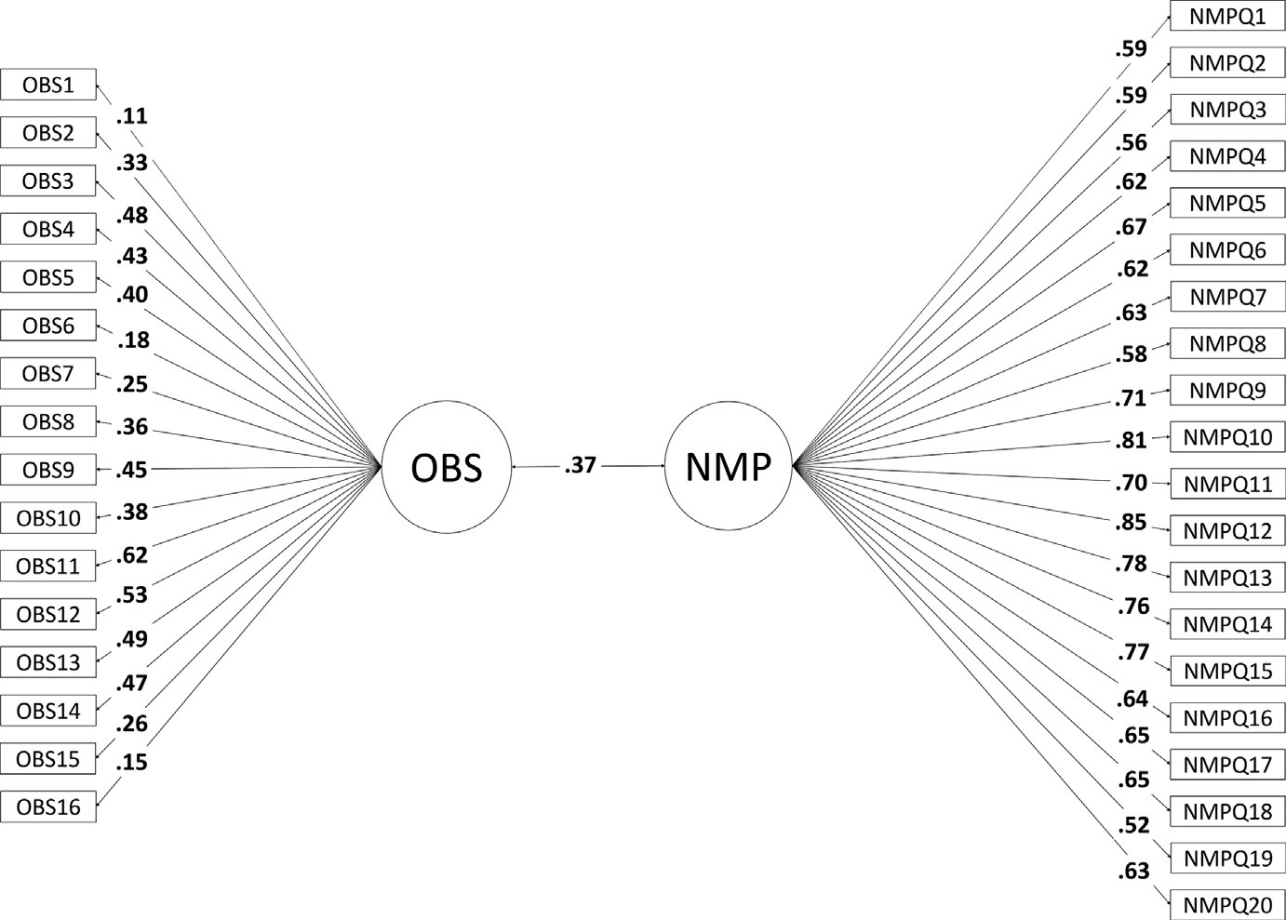
Structure Equation Model (SEM) of Obsessiveness and Nomophobia. This model (i.e., Model 1) consists of two latent factors: One for OBS and one for NMP-Q.

**Fig. 2:**
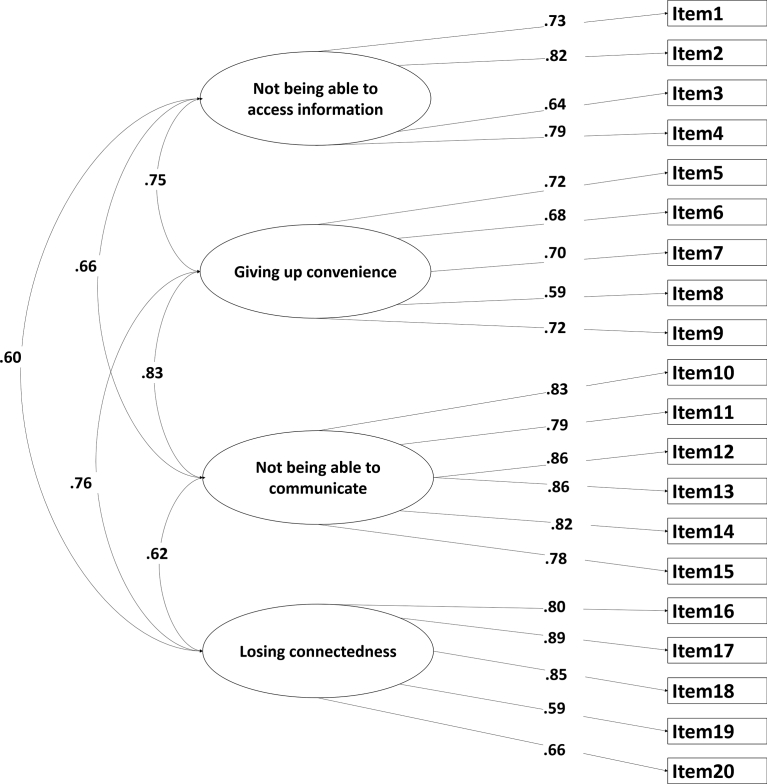
Confirmatory Factor Analysis (CFA) of Four Nomophobia Factors. This model (i.e., Model 2) consists of one for OBS and four factors of the NMP-Q as suggested by Yildirim and Correia (2015). The four factors of the NMP-Q were labelled as follows: “Not being able to communicate” (NMPQ_F1), “losing connectedness” (NMPQ_F2), “not being able to access information” (NMPQ_F3), and “giving up convenience” (NMPQ_F4). Correlation among the five constructs were examined.

In both models (see Figs. 1 and 2), data from the OBS Content Scale and NMP-Q were used as continuous variables. In preliminary analyses, fit indices of one factor model after loading all items of the OBS Content Scale and the NMP-Q (χ^2^ (594) = 2367.094, p < 0.01, CFI = 0.689, RMSEA = 0.087 (.083 - .090), SRMR = 0.08) did not indicate a good fit. Thus, two CFA models were tested to explore the relationship between the OBS Content Scale and NMP-Q. Model 1 consisted of two latent factors: one for OBS Content Scale and one for NMP-Q. Model 2 consisted of one for OBS Content Scale and four factors for the NMP-Q as suggested by Yildirim and Correia (2015). The four factors of the NMP-Q were labeled as “not being able to communicate” (NMPQ_F1), “losing connectedness” (NMPQ_F2), “not being able to information” (NMPQ_F3), and “giving up convenience” (NMPQ_F4). Correlations among the five constructs were examined. The chi-square difference test was utilized to see the model improvement between Model 1 and Model 2. A significant χ^2^ difference indicates a difference of fit between the two models.

Convergent validity was assessed using Average Variance Extracted (AVE), which represents the average amount of variance measured by indicator and Composite Reliability (CR) commonly recognized as less biased reliability measures than coefficient alpha. AVE values above 0.5 or CR values above of 0.7 are considered as acceptable evidences of convergent validity (Fornell and Larcker, 1981).

Divergent validity was assessed using the Heterotrait-monotrait ratio of the correlations (HTMT) approach (Henseler et al., 2015). If the value of the HTMT is higher than a proposed threshold, there is a lack of divergent validity. Kline (2011) suggested a threshold of 0.85, whereas others proposed a value of 0.90 (Teo et al., 2008).

## 7 Results

### 7.1 Checking measure assumptions

As shown in Table 1, no problematic feature was found from the measured psychometric properties. According to the skewness and kurtosis values for the measures fall under the recommended range of skewness and kurtosis (<3 and <10, respectively) by Kline (2011). The Bartlett's test of sphericity was significant (χ^2^ (190) = 5025.907, *p* < .01) which rejected the null hypothesis that the matrix was an identity matrix and the correlations in the correlation matrix were zero. The measured Kaiser-Meyer-Olkin (KMO) index which measures sampling adequacy, was .946 and thus is greater than the minimum acceptable value of .600 (Tabachnick and Fidell, 2013).

**Table 1:** Skewness, kurtosis, and reliability of the sample.

Total (N = 397)	Skewness	Kurtosis	Reliability, α
OBS	−.05 (−.17)	−.47 (−.51)	.72 (.73)
NMP-Q_F1	−0.57	−0.2	0.83
NMP-Q_F2	0.05	−0.68	0.81
NMP-Q_F3	−0.39	−0.62	0.93
NMP-Q_F4	0.46	−0.45	0.86
NMP-Q_All	−0.1	−0.39	0.94

*Note.* () two items of obsessiveness were dropped due to the low item-total correlation below .3.

Table 2 shows Pearson correlation coefficients among the factors. The four NMP-Q latent constructs (NMP-Q_F1-4) indicated strong effect sizes (r's ranged from 0.59 to 0.73) each other. Nevertheless, the effect sizes between OBS and NMP-Q_F1-4 were small to moderate (r's ranged from 0.16 and 0.33) and the four correlations were significant at p < 0.01.

**Table 2:** Pearson correlation coefficients among factors.

	2	3	4	5	6
1. OBS	.16**	.33***	.27***	.25***	.30***
2. NMP-Q_F1	-	.62***	.59***	.56***	.79***
3. NMP-Q_F2		-	.73***	.67***	.89***
4. NMP-Q_F3			-	.59***	.89***
5. NMP-Q_F4				-	.83***
6. NMP-Q_All					-

***p < .001, **p < .01, *p < .05.

### 7.2 Confirmatory factor analysis (CFA)

The first model (see Fig. 1) assessed whether a single NMP-Q and a single OBS Content Scale factor would best fit the data. While this model indicated a positive relationship with a moderate effect size (*r* = 0.37; Cohen, 1988), it was not a good fit as indicated by all fit indices except SRMR, χ^2^ (526) = 1829.120, p < 0.01, CFI = 0.770, RMSEA = 0.079 (.075–.083), SRMR = 0.06.

The second model (see Fig. 2) assessed the relationship between a four-factor NMP-Q model and the OBS Content Scale. The second model was a good fit to the data as indicated by most the fit indices, χ^2^ (517) = 1000.143, p < 0.01, CFI = 0.915, RMSEA = 0.049 (.044–.053), SRMR = 0.05. However, although the χ^2^ statistic was significant, usually indicating that the model did not capture all the variance in the data, models with large numbers of observations are often significant. Furthermore, the second model was a better fit than the first model, Δχ^2^ = 828.977, Δdf = 9, *p* < 0.01. In addition, 11.45% (=43.53–32.08) of increased variance could be found in the rotation sums of squared loadings (Table 3).

**Table 3:** Eigen values and total variance explained by factors before and after rotation.

Model	Factor	Initial eigenvalues			Rotation sums of squared loadings		
		Total	% of Variance	Cumulative %	Total	% of Variance	Cumulative %
Two factor model	NMP-Q	10.07	29.61	29.61	5.79	17.03	17.03
	OBS	2.89	8.50	38.10	5.12	15.05	32.08
Five factor model	NMP-Q_F3	10.07	29.61	29.61	4.87	14.31	14.31
	NMP-Q_F4	2.89	8.50	38.10	3.31	9.73	24.04
	NMP-Q_F1	1.80	5.29	43.40	2.85	8.38	32.43
	OBS	1.41	4.15	47.55	2.68	7.89	40.31
	NMP-Q_F2	1.21	3.56	51.11	1.09	3.22	43.53

*Note:* Extraction Method: Maximum Likelihood; Rotation Method: Varimax with Kaiser Normalization.

### 7.3 Convergent validity

We used AVE and CR to assess convergent validity. In the final model (the five-factor solution), we found acceptable AVE values only for NMP-Q_F3 (.56). Regarding CR, all factors showed CR above the acceptable threshold except for NMPQ-2 (see Table 4). These findings provide mixed support for convergent validity.

**Table 4:** Average variance extracted (AVE) and composite reliability (CR).

Model	Factor	AVE	CR
Two factor model	OBS	0.22	0.79
	NMP-Q	0.67	0.95
Five factor model	OBS	0.17	0.72
	NMP-Q_F1	0.44	0.75
	NMP-Q_F2	0.16	0.44
	NMP-Q_F3	0.56	0.88
	NMP-Q_F4	0.45	0.80

### 7.4 Divergent validity

We used HTMT to assess the discriminative validity of four factors (NMP-Q F1-4) mutually. As seen in Table 5, there was no value over .85 among four factors of NMP-Q, indicating evidence for divergent validity.

**Table 5:** Heterotrait-monotrait (HTMT) ratio of the correlations among NMP-Q_F1-4.

	2	3	4
1. NMP-Q_F1	.765	.673	.663
2. NMP-Q_F2	-	.839	.805
3. NMP-Q_F3		-	.661
4. NMP-Q_F4			-

## 8 Discussion

The present study closely examined the factor structure of the NMP-Q and its relationship with the OBS Content Scale in a sample of US college students. The study provided novel information regarding the convergent validity of five factors (NMPQ-F1, NMPQ-F2, NMPQ-F3, NMPQ-F4, and OBS Content Scale, see Table 4) and divergent validity of four dimensions of the NMP-Q (see Table 5).

We found moderate-sized relationships between the NMP-Q and the OBS Content Scale. In the first CFA model, a positive relationship was found between the average NMP-Q score and the average OBS Content Scale score. This relationship indicated that an obsessive individual tends to display more anxiety from not having a mobile phone (or vice versa). However, this model had a poor fit to the data. The second model exhibited a significantly better fit to the data than the first model and revealed a more nuanced relationship between obsessiveness and the four subcomponents of nomophobia. Fear of “giving up convenience” and “not being able to communicate” had moderate correlations with obsessiveness. The fear of “not being able to access information” and “losing connectedness” had small correlations with obsessiveness. The correlations found between obsessiveness, losing connectedness, and not being able to communicate may be explained by the need to stay connected in order to maintain social capital (see Model 2), in other words, maintaining relationships by keeping in contact (Tan et al., 2018). Tan et al. (2018) demonstrated that some personality traits are more inclined to use their mobile devices to maintain their social relationships (those with extroverted traits) while others may find mobile devices useful in terms of gaining social relationships (those with neurotic traits). The correlation between obsessiveness, giving up convenience, and not being able to access information is in line with findings of previous literature suggesting that heavy cell phone users lack the accuracy of concentration. The researchers ascribed this behavior to the conveniences that cellphone provide. As cellphones are almost always present, it leaves individuals with little need to have to concentrate or memorize bits of information (Abramson et al., 2009).

However, unlike previous research, we make the distinction between “personality traits” and “personality disorders.” Big 5 personality traits, such as agreeableness or conscientiousness, are considered as a characteristic that is static, whereas obsessiveness is classified either as a mental disorder or a psychological symptom that is malleable. According to King et al. (2013), nomophobia refers to a “situational” fear of being out of mobile phone contact and not being able to use it (King et al., 2013). This distinction is important because it implies that nomophobia can be treated or cured if diagnosed properly.

In regard to convergent validity, we found the adequate convergence of the five-factor model (NMP-Q_F1-4 and the OBS Content Scale—See Tables 2 and 4) based on the information provided by the CR values. These findings suggest that each of the questions within each factor showed a high degree of shared variance. One exception was the second factor of the NMP-Q (NMP-Q_F2), which had the low factor loadings (see Table 6) the lowest reliability estimates (as measured using both Cronbach's alpha), and the lowest convergent validity (as measured using both AVE and CR). These low scores differ from Yildirim and Correia (2015) and may have been caused by the different extracting methods used in this study (Maximum Likelihood CFA vs. PCA). The AVE scores for all variables were rather low (i.e., similar to or below the recommended cut-off of 0.50). At first blush, this might seem like evidence against convergent validity. However, according to recent recommendations of Henseler et al. (2015), AVE values are not always reliable because they do not rely on inference statistics, so there is no way to statically test for discriminant validity up to date. Interestingly, although the two-factor model of the NMP-Q and OBS Content Scale was not a good fit, the overall NMP-Q showed an AVE value above the recommended criteria, further supporting the cautious interpretation of those values. Thus, when considered together with the CFA analysis and the CR values, the majority of evidence supports the idea of convergent validity within each of the NMPQ-factors and the OBS Content Scale.

**Table 6:** Estimated factor loadings for five factors.

Item	Factor				
	OBS	NMP-Q_F1	NMP-Q_F2	NMP-Q_F3	NMP-Q_F4
OBS2	0.342				
OBS3	0.459				
OBS4	0.392				
OBS5	0.391				
OBS6	0.210				
OBS7	0.240				
OBS8	0.378				
OBS9	0.428				
OBS10	0.367				
OBS11	0.615				
OBS12	0.522				
OBS13	0.467				
OBS14	0.473				
OBS15	0.253				
NMPQ1		0.632			
NMPQ2		0.797			
NMPQ3		0.480			
NMPQ4		0.693			
NMPQ5			0.596		
NMPQ6			0.524		
NMPQ7			0.356		
NMPQ8			0.210		
NMPQ9			0.124		
NMPQ10				0.708	
NMPQ11				0.803	
NMPQ12				0.723	
NMPQ13				0.832	
NMPQ14				0.756	
NMPQ15				0.668	
NMPQ16					0.730
NMPQ17					0.843
NMPQ18					0.772
NMPQ19					0.464
NMPQ20					0.466

*Note.* Extraction Method: Maximum Likelihood, Rotation Method: Varimax with Kaiser Normalization.

In regard to divergent validity, we showed strong evidence that each of the four factors of the NMP-Q differed from one another. Specifically, the HTMT ratio of the correlations between each of the NMP-Q factor surpassed the recommended cut-offs of 0.70. These findings are consistent with the five factor-model of the CFA analysis that showed a better fit than the two-factor model that combined all of the NMP-Q questions. Thus, the underlying structure of the NMP-Q is consistent with the original EFA conducted by Yildirim and Correia (2015) and should be considered a set of related, but sufficiently different underlying factors. Supporting this conclusion, a recent study showed that only two of the four NMP-Q scales were negatively correlated with quiz performance after being distracted by their mobile phone during a video lecture (Mendoza et al., 2018).

The psychometric investigation of the NMP-Q can be useful in assisting clinicians, and other professionals in obtaining a more nuanced vantage point on how to characterize nomophobia. According to Bragazzi and Del Puente (2014), nomophobia “is the pathological fear of remaining out of touch with technology” (p. 156). With this in mind, researchers believe that nomophobia may act serve as a proxy for identifying other psychiatric diseases that share similar symptoms to those associated with nomophobia (Bragazzi and Del Puente, 2014). The present study reinforces this idea by improving the understanding between pre-existing assessments of personality disorders (e.g., obsessiveness) that are surfacing from mobile phone related phobia.

Individuals who show greater obsessive behavior (such as checking one's pockets every ten seconds to see what is going on) will have moderately more anxiety from “not being able to communicate with others” and/or “giving up convenience.” As a result, we infer that this relationship will show a possible increase in anxiety from losing connectedness and access to information via their cellphones.

The research has defined nomophobia as fear and subset of anxiety that arises from not having access to one's mobile device. With characteristics that resemble anxiety, (i.e., impulsivity, unreasonable and irrational fears of losing connectedness), it is important to discuss the clinical applications of understanding this behavioral dependence. Bragazzi and Del Puente (2014) point out it is common for psychiatric diseases to cluster into similar categories. For example, anxiety may be grouped together in a similar category to panic disorder, and social phobia. Similarly, the obsessive-compulsive disorder may be grouped with eating disorders, depression, alcohol and/or drug addiction disorders (Bragazzi and Del Puente, 2014).

The current study has several limitations. While the study sample size was sufficiently large, participants were selected via convenience sampling (i.e., university students within psychology courses). In terms of external validity, the generalizability of the current findings to a larger population is somewhat limited. For example, younger generations might be more susceptible to nomophobia than older generations who did not grow up with such technology. Future studies can further generalize these findings by using different sampling methods. The current study also did not assess biological or environmental factors such as gender and culture that might moderate these relationships. For example, women have higher rates of nomophobia than men (Yildirim et al., 2016), whereas men are more likely to show an earlier onset of obsessive-compulsive disorder (Bogetto et al., 1999; Lensi et al., 1996). Another limitation is that the data were collected via self-report measures, which may weaken the construct validity of this study, as some researchers questioned whether self-report measures are comparable across different groups like (Gregorish, 2006; Lin and Pakpour, 2017).

## 9 Conclusion

The present study supports the NMP-Q as a promising measure that has relevance to educators and healthcare professionals. As Lin and Pakpour, 2017 supported, the properties of the NMP-Q were “robust.” Out study also shows that the latent structure of the NMP-Q is better represented by multiple sub-factors that have a high degree of shared variance within each sub-factor but are divergent from one another. Although collecting more clinical diagnoses are needed, we found that the NMP-Q has a meaningful comparison across obsessive behavior. Future research is still encouraged to examine the psychometric properties of the NMP-Q across other psychological concepts (e.g., internet addiction) due to the limitations of our study.

The higher scores of obsessiveness corresponded to higher severity levels of nomophobia, which might contribute to growing clinical symptoms based on new technological developments. This relationship suggests, for example, that being without one's mobile phone might lead to habitual distraction in everyday situations such as classroom learning, heightened state of anxiety, and poor short-term memory (e.g., Cheever et al., 2014; Lee et al., 2017; Mendoza et al., 2018; Sparrow et al., 2011). Specifically, being without one's phone may lead to distracting thoughts on what messages or news may be awaiting. The study provides a valuable insight to incorporate four dimensions of nomophobia in clinical research and practice using the self-reported measure (i.e., the NMP-Q).

From a mental health standpoint, a psychometric investigation on the NMP-Q is needed in order to verify its use as a proxy for helping clinicians identify comorbidity of disorders as smartphone usage has become an essential part of individual's life. Additionally, psychological disorders are not always clear upon preliminary diagnoses, and it can be easy to dismiss disorders when only looking at individual's behavior alone. If clinicians begin to use phone dependency as a means to verify what individuals are doing on their phone and the extent to which their phone use influences their behaviors, it may serve as a highly useful tool in not only identifying psychological disorders but also in identifying maladaptive behaviors that can be modified through clinical therapeutic methods. Further research is needed on this to provide more support on this argument.

## Declarations

### Author contribution statement

Seungyeon Lee: Conceived and designed the experiments; Wrote the paper.

Minsung Kim: Conceived and designed the experiments; Analyzed and interpreted the data.

Jessica S. Mendoz: Performed the experiments; Wrote the paper.

Ian M. McDonough: Performed the experiments; Wrote the paper.

### Funding statement

This research did not receive any specific grant from funding agencies in the public, commercial, or not-for-profit sectors.

### Competing interest statement

The authors declare no conflict of interest.

### Additional information

No additional information is available for this paper.
